# Case Report: Successful treatment of pityriasis rubra pilaris with deucravacitinib

**DOI:** 10.3389/fimmu.2026.1752884

**Published:** 2026-02-04

**Authors:** Yixuan Li, Meiliang Guo, Ziyao Sheng, Zhehong Zhou, Na Liu, Qinqin Meng, Hui Deng

**Affiliations:** Department of Dermatology, Shanghai Sixth People’s Hospital Affiliated to Shanghai Jiao Tong University School of Medicine, Shanghai, China

**Keywords:** deucravacitinib, inflammatory skindisease, pityriasis rubra pilaris, treatment, TYK2 inhibitor

## Abstract

Pityriasis rubra pilaris (PRP) is a rare inflammatory skin disease characterized by hyperkeratotic follicular papules, palmoplantar hyperkeratosis, and associated normal “islands of unaffected skin”. Its pathogenesis has not been fully elucidated, and treatment poses significant challenges. Conventional therapies include oral retinoids and topical emollients. In recent years, although biological agents have been used in treatment, they are associated with side effects such as an increased risk of infection, and some patients show no response to treatment, thus necessitating the exploration of new therapeutic approaches.This case represents the first reported use of a TYK2 inhibitor (deucravacitinib) for the treatment of PRP. The patient was a 39-year-old male who developed extensive erythema in December 2024. The erythema gradually increased and progressed to red punctate eruptions accompanied by mild desquamation, slight pruritus with a stinging sensation, and “islands of unaffected skin”. Initial treatment with topical dinoprostone and mometasone furoate cream was ineffective. In March 2025, the patient received deucravacitinib (6 mg daily) in combination with topical halometasone cream. At the 1-month follow-up, significant improvements were observed in erythema, desquamation, and pruritus. At the final 6-month follow-up, the skin lesions almost resolved, leaving only mild erythema and a small amount of desquamation. Both the disease severity and the patient’s quality of life were significantly improved. This case suggests that deucravacitinib exhibits favorable efficacy and safety in the treatment of PRP. However, due to the low incidence of PRP, which makes large-scale controlled trials difficult, and the lack of recognized treatment guidelines, more clinical studies are needed in the future to further verify the potential of deucravacitinib in the treatment of PRP.

## Introduction

Pityriasis rubra pilaris (PRP) is a rare inflammatory skin disorder characterized by hyperkeratotic follicular papules, palmoplantar hyperkeratosis, and well-demarcated orange-red scaly plaques with intervening normal “islands of sparing” (1). Due to its incompletely understood pathogenesis, treatment remains a significant challenge. Traditional therapies include oral retinoids (1) and topical emollients to alleviate symptomatic discomfort. Recent studies have implicated dysregulation of the IL-23/Th17 axis in PRP pathogenesis (2), providing a theoretical basis for the use of biologics such as ustekinumab and secukinumab in PRP treatment. Additionally, Janus kinase (JAK) inhibitors and phosphodiesterase-4 (PDE4) inhibitors have shown promising efficacy in PRP management (3–5). However, the potential side effects of biologics and small-molecule inhibitors, including increased infection risk, cannot be overlooked. Moreover, some patients remain refractory to these treatments, necessitating the exploration of novel therapeutic options. We report the first case of significant improvement in PRP skin lesions using deucravacitinib, a TYK2 inhibitor.

**Figure 1:**
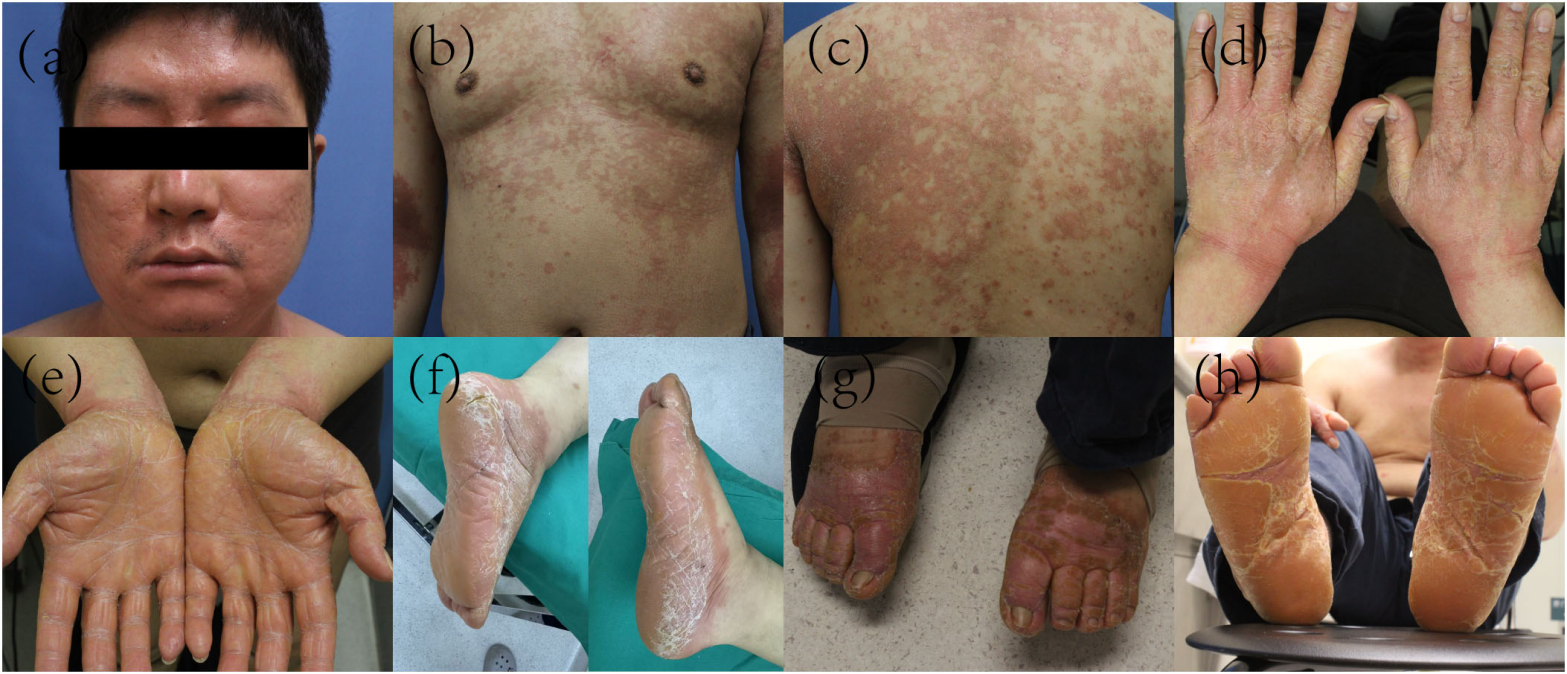
Systemic PRP leisions before the initiation of treatment. The images show the face, chest and back of the patient with histologically confirmed pityriasis rubra pilaris at initial presentation with follicular papules, scaly red-orange patches **(a–c)**. Additionally, hyperkeratotic plaques are observed on the bilateral palms, the dorsum of the hands **(d, e)**, as well as the dorsal and plantar feet **(f–h)**.

**Figure 2:**
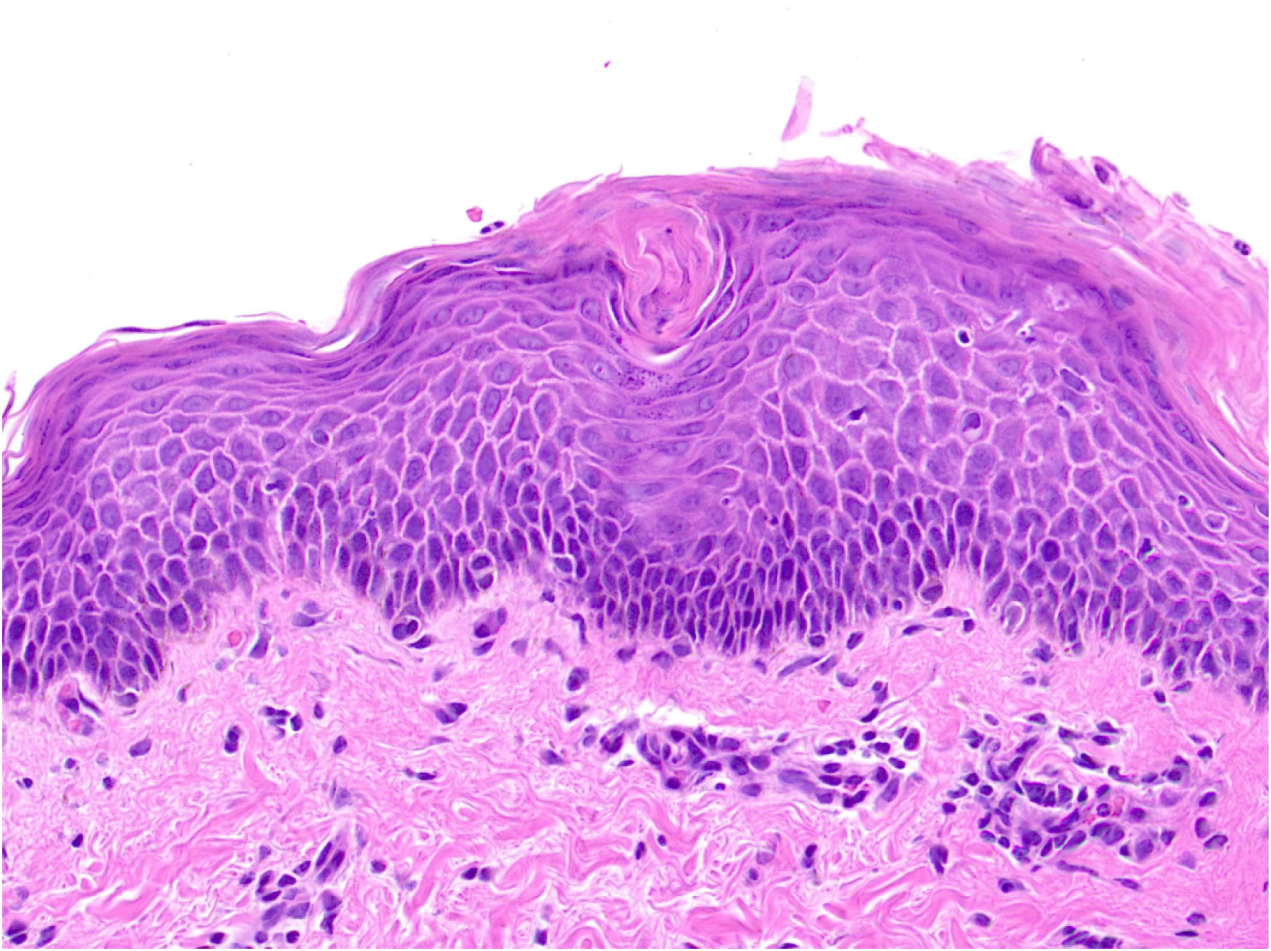
Histopathological examination showed squamous epithelial hyperplasia with parakeratosis, elongated rete ridges, edema in the papillary dermis, and perivascular infiltration of numerous chronic inflammatory cells and the presence of cutaneous horny plugs.

**Figure 3:**
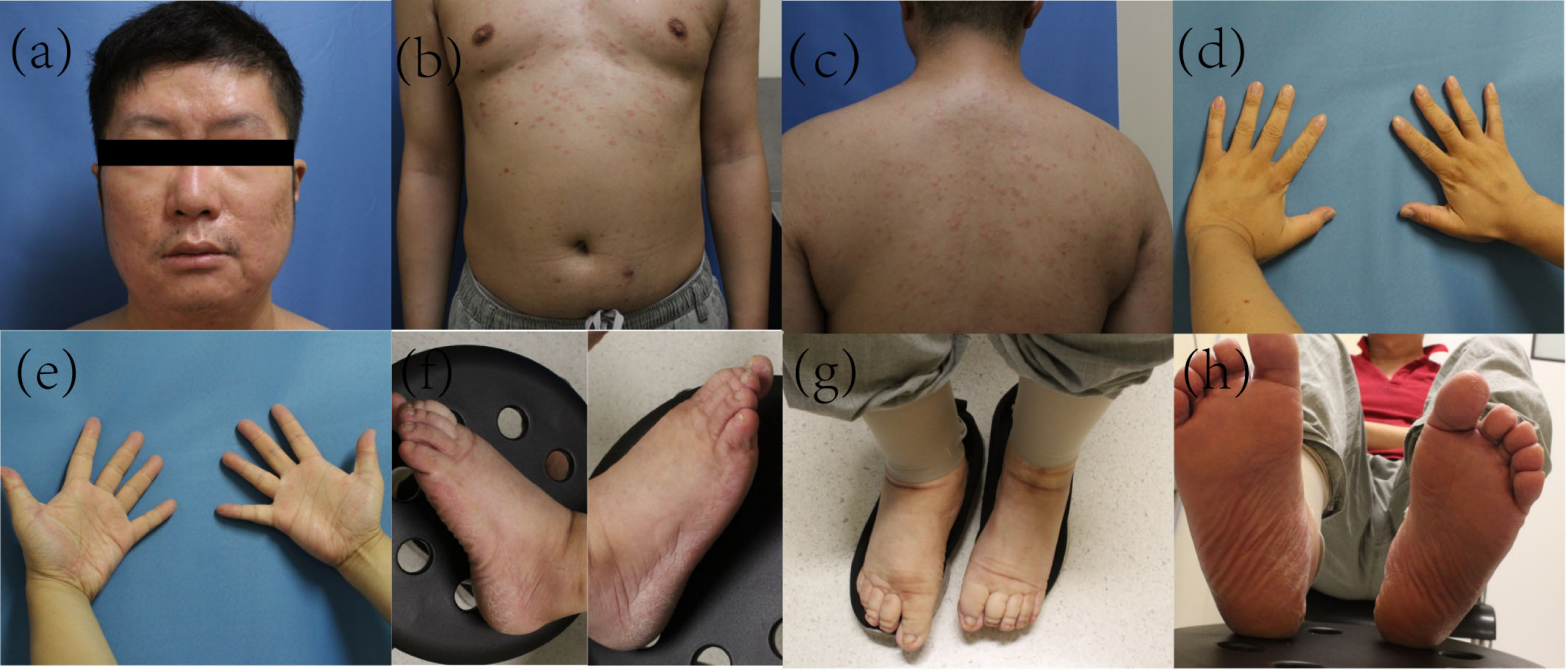
Systemic PRP leisions after 6 months of treatment. At months 7 of deucravacitinib therapy, the erythema on the face, anterior and posterior chest was significantly relieved **(a–c)**, and the hyperkeratosis on both hands and feet was markedly improved **(d–h)**.

The patient was a 39-year-old male with a history of hypertension. In December 2024, he developed a large erythematous patch that gradually increased in number, accompanied by mild scaling and slight prickling pruritus, which later progressed to red punctate rashes with expanding lesion areas. Physical examination revealed generalized erythema and scaly plaques with follicular hyperkeratosis affecting the trunk, abdomen, limbs, face, and scalp, with visible “islands of sparing” (**Figure 1**). Initially, he was diagnosed with pityriasis rosea, but treatment with dinoprostone combined with mometasone furoate cream for one month yielded no significant improvement. To further confirm the diagnosis, a pathological section was performed for him.Histopathological examination showed squamous epithelial hyperplasia with parakeratosis, elongated rete ridges, edema in the papillary dermis, and perivascular infiltration of numerous chronic inflammatory cells and the presence of cutaneous horny plugs (**Figure 2**). Initial treatment with topical dinoprostone and mometasone furoate cream yielded no significant improvement. Based on histological findings and clinical presentation, a diagnosis of pityriasis rubra pilaris was favored, with the specific subtype being typical adult-type pityriasis rubra pilaris. After taking the medical history, it was found that the patient was infected with COVID-19 in December 2022. In addition, a previous literature has reported a case of pityriasis rubra pilaris following COVID-19 infection, which may suggest a potential association with the onset of the disease (6). The patient had no other specific infection history or relevant family history prior to the onset of skin lesions. Previous studies have indicated that the efficacy of acitretin varies from person to person. A systematic review conducted by Kromer et al. showed that only 43 out of 174 pityriasis rubra pilaris (PRP) patients achieved favorable efficacy after receiving acitretin treatment (7).Considering that deucravacitinib is non-immunogenic, which reduces the production of anti-drug antibodies and thus ensures more stable long-term therapeutic efficacy. Meanwhile, deucravacitinib selectively inhibits tyrosine kinase 2 (TYK2) without exerting inhibitory effects on other Janus kinases, thereby decreasing the incidence of adverse reactions. In addition, deucravacitinib has shown favorable efficacy in improving psoriasis in special sites such as palms, soles, scalp and nails. Therefore, we recommend deucravacitinib for the treatment of the patient. On March 4, 2025, the patient began treatment with deucravacitinib (6 mg daily), combined with topical halometasone cream. At the first follow-up (1 month), significant improvements in erythema, scaling, and pruritus were observed. The patient continued deucravacitinib therapy. By the final visit to our hospital (6 months), his lesions had nearly resolved, with only mild erythema and minimal scaling remaining (**Figure 3**). The Psoriasis Area and Severity Index (PASI) score decreased from 33.5 to 2.6, and Dermatology Life Quality Index (DLQI) score improved from 23 to 4, indicating substantial improvements in both disease severity and quality of life. These results demonstrate the significant potential of our treatment regimen in managing PRP.

Similar to other rare diseases, the low incidence of PRP makes large-scale controlled trials challenging, complicating the development of widely accepted, effective treatment guidelines. Currently, systemic retinoids are the most commonly used treatment. In recent years, case reports have documented the use of biologics and small-molecule inhibitors; however, their potential side effects, such as increased infection risk, remain a concern.

**Figure 4:**
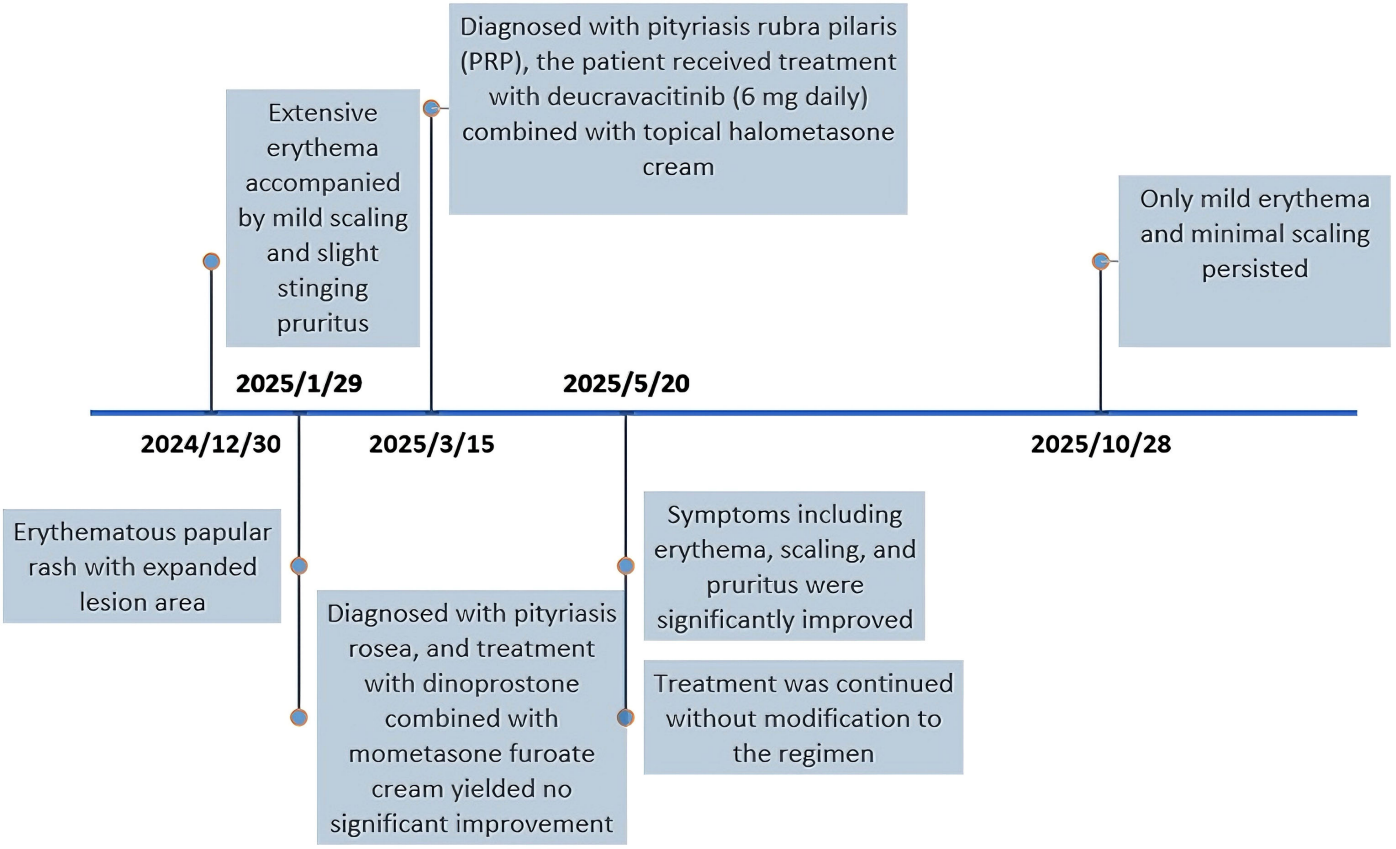
Case timeline.

Pityriasis rubra pilaris is classified into six subtypes: Classical adult (I), Atypical adult (II), Classical juvenile (III), Circumscribed juvenile (IV), Atypical juvenile (V), HIV-related (VI), In this case, the patient is classified as Type I, it’s the commonest type (55% of cases), cephalocaudal progression, ‘suberythrodermia’ with nappes claires, palmoplantar keratoderma (1). This case represents the first report of a TYK2 inhibitor used to treat PRP. The timeline of onset, diagnosis and treatment for this patient is shown in **Figure 4**. We observed that PRP shares significant genetic and immunological overlap with psoriasis (8), as well as similarities in clinical and histopathological features. Studies have shown that TYK2 inhibitors bind to the regulatory domain of TYK2 (a Janus kinase), disrupting IL-23, IL-12, and type I interferon signaling pathways, which are considered critical in psoriasis pathogenesis (9). TYK2 inhibitors, including deucravacitinib, have demonstrated a favorable safety profile. Deucravacitinib has shown remarkable efficacy in psoriasis treatment, effectively alleviating symptoms, reducing clinical manifestations, and significantly improving patients’ quality of life (10). The drug has also recently received FDA approval for moderate-to-severe psoriasis. Our study highlights the efficacy and safety of deucravacitinib in PRP treatment. Nowadays, the patient is still receiving continuous medication. During the follow-up period, the patient’s condition has remained stable without signs of recurrence, but data regarding the disease progression and potential recurrence risk after drug withdrawal are still lacking. In subsequent research, continuous follow-up of this case will be conducted to further improve the relevant data. Moreover, future clinical trials are needed to further investigate its therapeutic potential and safety profile in PRP management.

## Data Availability

The original contributions presented in the study are included in the article/supplementary material, further inquiries can be directed to the corresponding authors.

## References

[1] Roenneberg S Biedermann T . Pityriasis rubra pilaris: algorithms for diagnosis and treatment. J Eur Acad Dermatol Venereol. (2018) 32:889–98. doi: 10.1111/jdv.14761, PMID: 29247481

[2] Potestio L D’Agostino M Portarapillo A Esposito V Tommasino N Salsano A . Emerging role of biologic drugs targeting IL-17 and IL-23: pityriasis rubra pilaris. Life (Basel). (2024) 14:923. doi: 10.3390/life14080923, PMID: 39202665 PMC11355122

[3] Inoue E Arase N Hanaoka Y Tanemura A Fujimoto M . The beneficial effect of a PDE4 inhibitor in a patient with juvenile-onset intractable pityriasis rubra pilaris without CARD14 mutation. Dermatol Ther. (2021) 34:e14714. doi: 10.1111/dth.14714, PMID: 33368948

[4] Saad M Spurr A Lipson J . Pityriasis rubra pilaris partially responsive to treatment with upadacitinib: A case report. SAGE Open Med Case Rep. (2023) 11:2050313X231160927. doi: 10.1177/2050313X231160927, PMID: 37009550 PMC10064474

[5] Tan H Zhang B Kang X Wang L Qiu X Hu X . Tofacitinib for pityriasis rubra pilaris: A case report. Clin Cosmet Investig Dermatol. (2024) 17:1917–20. doi: 10.2147/CCID.S470170, PMID: 39220289 PMC11363948

[6] Aromolo IF Pisapia A Riva D Barberi F Marzano AV Moltrasio C . COVID-19 induced pityriasis rubra pilaris: A superantigenic disease? J Eur Acad Dermatol Venereol. (2023) 37:e26–8. doi: 10.1111/jdv.18556, PMID: 35993491 PMC9538733

[7] Kromer C Sabat R Celis D Mössner R . Systemic therapies of pityriasis rubra pilaris: a systematic review. J Dtsch Dermatol Ges. (2019) 17:243–59. doi: 10.1111/ddg.13718, PMID: 30520557

[8] Fuchs-Telem D Sarig O van Steensel MA Isakov O Israeli S Nousbecket J . Familial pityriasis rubra pilaris is caused by mutations in CARD14. Am J Hum Genet. (2012) 91:163–70. doi: 10.1016/j.ajhg.2012.05.010, PMID: 22703878 PMC3397268

[9] Kingston P Blauvelt A Strober B Armstrong AW . Deucravacitinib: a novel TYK2 inhibitor for the treatment of moderate-to-severe psoriasis. J Psoriasis Psoriatic Arthritis. (2023) 8:156–65. doi: 10.1177/24755303231201336, PMID: 38188537 PMC10768812

[10] Armstrong AW Gooderham M Warren RB Strober B Thaçi D Sofen H . Deucravacitinib versus placebo and apremilast in moderate to severe plaque psoriasis: Efficacy and safety results from the 52-week, randomized, double-blinded, placebo-controlled phase 3 POETYK PSO-1 trial. J Am Acad Dermatol. (2023) 88:29–39. doi: 10.1016/j.jaad.2022.07.002, PMID: 35820547

